# The role of schools in mental health promotion and prevention: a document analysis of Swedish local mental health policies

**DOI:** 10.1093/heapro/daag053

**Published:** 2026-04-17

**Authors:** Lisa Rydstad, Camilla Eriksson, Peter Larm, Maria Fjellfeldt

**Affiliations:** Department of Public Health Sciences, Stockholm University, 106 91, Stockholm, Sweden; Department of Health Sciences, Innovation and Design, Mälardalen University, Box 883, 721 23, Västerås, Sweden; Department of Public Health Sciences, Stockholm University, 106 91, Stockholm, Sweden; Department of Health and Welfare, Dalarna University, 791 88, Falun, Sweden

**Keywords:** mental health, school health promotion, document analysis, children, adolescents, health-promoting schools

## Abstract

It has been recognized that schools play a key role in promoting well-being and preventing mental health problems among children and adolescents. The local context is crucial for realizing the school’s health-promotive potential. This study aimed to explore how the role of schools is approached in Swedish municipal policy documents governing mental health efforts. A qualitative document analysis using the READ approach was conducted. Seventy-three municipalities were selected and asked to provide policy documents on mental health; twelve policy documents met the inclusion criteria and were analyzed using conventional content analysis. The analysis identified the school as a central actor across the documents. Four categories were constructed: (i) the school as a context influencing mental health, (ii) the school as a platform for reaching all children and adolescents with mental health interventions, (iii) the school as responsible for detecting mental health problems, and (iv) the school as a collaborating partner in mental health efforts. The analysis highlighted a primarily individualized and often medicalized understanding of mental health problems, positioning health professionals as key actors. Although the school’s role as a context influencing mental health was clearly acknowledged, teachers, pedagogical support and social relationships were overlooked in relation to this role. The school’s role in mental health efforts was thus largely detached from its educational mission. Finally, there was substantial variation in the activities expected of schools across municipalities. Hence, despite schools’ recognized importance, their role in mental health efforts was subject to local interpretation of national policy within a decentralized school system.

Contribution to Health Promotion

- This study provides insights into how the school’s role is approached in Swedish municipal policy documents governing mental health efforts.
- The analysis highlights a primarily individualized and often medicalized understanding of mental health problems.
- School health professionals were key actors, delivering universal school-based mental health interventions and referring students to treatment.
- Teachers, pedagogical support, and social relationships were largely overlooked when schools were conceptualized as a context influencing mental health.
- The school’s role in mental health efforts was largely detached from its educational mission.

## Introduction

It has been recognized that schools play a key role in health promotion and prevention among children and adolescents. As the place where they spend most of their time outside the home, schools constitute both a major context influencing psychosocial development and an arena through which children and adolescents can be reached (World Health Organization 2018, 2024). As new public health challenges emerge, health-promotive and preventive efforts must be reviewed to ensure that actions in schools align with students’ health needs (Jourdan *et al*. 2021). In European high-income countries such as Sweden, there is a growing mental health crisis among children and adolescents (Tarasenko *et al*. 2025). Promoting well-being and preventing mental health problems in this group therefore constitutes a global public health challenge (World Health Organization 2024). Using the case of municipal policy documents governing mental health efforts in Sweden, this study explores how the role of schools is approached in responding to this challenge.

Mental health is a broad concept encompassing both mental well-being and mental health problems (Dalman *et al*. 2021). In children and adolescents, well-being can be understood as the ability to maintain a positive quality of life and reach expected psychological, social, and emotional developmental stages appropriate for their age (Blakemore 2019, Homman *et al*. 2025). Mental health problems include mental distress, milder difficulties such as worry, sadness, and sleep problems and mental disorders, which are more severe conditions requiring healthcare interventions (Dalman *et al*. 2021). Mental health problems that develop into mental disorders often begin early in life, with one-third starting before the age of 14 and half before the age of 18 (World Health Organization 2024). The school environment therefore offers a unique opportunity to promote positive mental health and prevent mental health problems during these critical years of childhood and adolescence (Weare and Nind 2011). Universal approaches target broad groups, such as entire schools. Selective prevention focuses on groups of students at higher-than-average risk, while indicated prevention supports individual students showing early symptoms. Mental health promotion, which is also universal and targets the whole school, aims to enhance overall well-being (Institute of Medicine Committee on Prevention of Mental Disorders 1994).

Mental health efforts in schools are situated within the broader concept of school health promotion (Jourdan *et al*. 2021, Hernández-Torrano *et al*. 2024). Traditionally, school health efforts emphasized health education, focusing mainly on the delivery of information from health professionals about risk factors associated with illness. From this traditional perspective, the school is approached as a platform for reaching all children through school-based health initiatives (Rowling and Jeffreys 2006). With the 1986 Ottawa Charter for Health Promotion (World Health Organization 1986), attention shifted toward the influence of living conditions on health, recognizing the complex interaction of factors that impact health within a system or organization. This gave rise to the settings-based approach, which views health as shaped by the broader social and organizational environments in which people live, learn, and work (Simovska 2012, Lindegaard Nordin *et al*. 2019, Karlander and Geidne 2025). Schools are understood not only as places where health knowledge is transmitted, but as settings in which organizational and personal dynamics interact to actively support and shape students’ health and well-being (Jourdan *et al*. 2021, Svensson and Warne 2024). Thus, within the settings-based approach, which highlights the reciprocal relationship between health and learning, offering a health-supporting and inclusive learning environment is regarded as a pathway to achieving educational goals (Jourdan *et al*. 2021, Hernández-Torrano *et al*. 2024, Karlander and Geidne 2025). The World Health Organization integrates these perspectives by advocating that health-promoting schools both address the social determinants of health within the school context and implement evidence-based health interventions (World Health Organization 2021).

In the school health promotion literature, two distinct approaches to mental health efforts can be identified (Hernández-Torrano *et al*. 2024, Svensson and Warne 2024). When the school is emphasized as a platform, the focus lies on school-based initiatives that directly address dimensions of mental health at the individual level. These initiatives are delivered by, or in collaboration with, school health services. School-based mental health initiatives are known by various terms, and there are thousands of interventions in the field, including social and emotional learning (SEL), mindfulness, resilience, life skills, and mental health awareness (Weare and Nind 2011, Foulkes *et al*. 2025). When the school is approached as a setting, the social determinants of mental health within the school context are addressed. These include, but are not limited to, peer relationships (Modin *et al*. 2018, Brolin Låftman *et al*. 2023), pedagogical practices (Bortes and Giota 2024, Bortes 2025), and pedagogical support (Östberg *et al*. 2015, Modin *et al*. 2017). From a settings-based approach, the promotion of well-being and the prevention of mental health problems are therefore considered to be closely related to the educational mission. It is thus not a task only for health professionals, but for everyone working in the school context (Rowling and Jeffreys 2006, Simovska 2012, Jourdan *et al*. 2021).

Policies, including local community strategies, are central to the implementation of school health promotion (Lindegaard Nordin *et al*. 2019). In the Swedish context, municipalities play a crucial role in interpreting and implementing national health and education policy (Elsayed 2025), as school governance is decentralized (The Swedish Association of Local Authorities and Regions 2025a). Although not directly targeting mental health promotion and prevention, previous Swedish studies show considerable variation in how municipalities interpret and organize national directives on broader school health promotion and prevention (Bergnehr and Johansson 2023, Elsayed 2025), possibly reflecting ambiguities in Swedish national policy and guidelines (Bergnehr and Gunnarsson 2025). A recent study examined how municipal governing documents addressed mental health promotion and prevention for children and adolescents. One of the main findings was that schools were positioned as primary actors in municipal mental health promotion and prevention, by implementing evidence-based programs, with a strong emphasis on psychoeducation (Fjellfeldt and Eriksson 2026). While there are many studies exploring the implementation of mental health policies in schools (e.g. Guerra *et al*. 2019, Teutsch and Gugglberger 2020), to our knowledge, no previous study has explored how the role of schools is approached in local mental health policies.

In Sweden, the rise in mental health problems among children and adolescents is a public health concern (The National Board of Health and Welfare 2024). However, research is lacking on how Swedish municipalities conceptualize and operationalize the role of schools in policies governing mental health efforts. Policy can be defined in many ways, depending on the research approach and the context in which it is applied (Hupe and Hill 2016). In this study, the focus is on examining what municipalities intend schools to do. Accordingly, the analysis centers on policy documents that provide clearly defined, prescriptive guidance (Hupe and Hill 2016), explicitly indicating the expected actions and responsibilities of schools in local mental health efforts.

### Aim

The aim of this study is to explore how the role of schools is approached in Swedish municipal policy documents governing mental health efforts.

## Methods

### Design

A qualitative research design was employed (Creswell and Creswell 2023), involving the collection and qualitative document analysis of policies using the READ approach (Dalglish *et al*. 2020). The READ approach provides a structured method for examining documents and can be applied to public health policy at the local level (Dalglish *et al*. 2020). Documents consisting of municipality-wide Swedish policies on mental health were examined, as they specifically target mental health efforts at the local level. As such, this type of document is well suited for exploring how the role of schools is conceptualized in municipal policies governing mental health efforts.

### The Swedish context

#### Mental health promotion and prevention in Swedish municipalities

Swedish municipalities are responsible for a wide range of local welfare services relevant to mental health promotion and prevention. These include compulsory school and high school, preschool and childcare, social services, services for persons with disabilities, elderly care, and physical planning. Municipalities have a voluntary responsibility for cultural and leisure activities for residents (The Swedish Association of Local Authorities and Regions 2025a). Given the many responsibilities, the Swedish national strategy on mental health and suicide prevention emphasizes municipalities as one of the key actors (Government Offices of Sweden 2025). Municipalities are not legally required to develop a local policy on mental health. As a result, the format of such documents is flexible, and initiatives to create the policy originate from the municipality itself.

#### Schools in Swedish mental health promotion and prevention

Schools are recognized as having a key role in Swedish mental health promotion and prevention. At the policy level, Sweden’s new 2025–34 national mental health strategy (Government Offices of Sweden 2025) and the National Public Health Policy (The Public Health Agency of Sweden 2022) highlight schools as central actors. Furthermore, health promotion is well integrated into the Education Act (Education Act 2010), which stipulates that elementary and high schools are responsible for implementing health-promoting and preventive measures provided by multidisciplinary school health services in collaboration with the principal and teachers. These measures should target health directly through universal, selective, and indicated interventions and simultaneously support educational achievement (Education Act 2010). Moreover, the curriculum for elementary school (The Swedish National Agency for Education 2024a) emphasizes the school’s role in fostering a safe, supportive learning environment and highlights that education should contribute to students’ personal development and well-being. Hence, safeguarding and promoting health is not a peripheral task in Swedish educational policy, but a core responsibility of the entire school involving teachers, school health services, and the principal.

#### The Swedish school system

In Sweden, compulsory education lasts from ages 6 to 16, while high school, for students aged 16 to 19, is voluntary, although the vast majority choose to continue. The Swedish school system is regulated at the national level through legislation, curricula, and policy frameworks established by the government and national authorities (Education Act 2010, The Swedish National Agency for Education 2024a). School governance is decentralized across 290 municipalities (The Swedish Association of Local Authorities and Regions 2025a). Municipalities are required to ensure that all children residing in the municipality have access to education and therefore operate municipal schools, although students may also attend schools operated by private providers. Education in Sweden is tuition-free regardless of school organizer (Education Act 2010, The Swedish Association of Local Authorities and Regions 2025a, 2025b). The majority of Swedish schools are operated by municipalities, one in five compulsory schools and one in three upper secondary schools are private (The Swedish National Agency for Education 2024b).

The principal in Swedish schools has a comprehensive responsibility, including pedagogical leadership, staff management, implementation of the national curriculum and education act, ensuring a safe and supportive learning environment, and coordinating the school health services. School health services are provided by physicians, school nurses, psychologists, counselors, and teachers with special educational competence. The school organizer, often the municipality, holds overall responsibility for ensuring that these services are available, although municipalities have discretion in how school health services are organized, meaning that staff may be centrally employed or placed at the school level under the principal’s authority. The work of the school health services should primarily be promotive and preventive in collaboration with teachers and school management (Education Act 2010). There are no national directives regulating responsibility for health education to groups. However, school nurses are required to meet individually with all children and adolescents for health dialogues, primarily focusing on health behaviors (Golsäter *et al*. 2012).

#### Mental health among Swedish children and adolescents

In Swedish self-reported surveys, mental health problems and psychosomatic symptoms among children and adolescents have increased markedly over the past two decades (Bremberg 2015, Potrebny *et al*. 2017, Hagquist *et al*. 2019, Grigorian *et al*. 2023). The prescription of antidepressant medications has risen alongside the increase in depression and anxiety disorders (The National Board of Health and Welfare 2024). Compared with other factors, such as relational issues and the home environment, school-related factors, such as school-related stress and performance demands, are among the main contributors to the rise in mental health problems in Sweden (Anniko *et al*. 2019, Löfstedt *et al*. 2020, Högberg 2021, 2024, Tillfors *et al*. 2024). The Swedish school system has undergone many changes since the 1990s, including decentralization and partial marketization of school governance, higher requirements for passing grades and high school eligibility, and earlier grading (Löfstedt *et al*. 2020, Högberg 2021, Högberg *et al*. 2021). These changes have occurred alongside a more uneven distribution of resources and support (Klapp *et al*. 2024, Högberg and Strandh 2025). Consequently, school-related stress and performance demands are consistently identified as some of the most important sources of mental health problems for children and adolescents in Sweden, particularly among girls (Högberg 2021, 2024, Tillfors *et al*. 2024).

### Sample and procedures

This study is part of a larger research program called Child Promotion/Prevention (ChiPP) for mental health outcomes—from policy to actions and their effects. The research program defined the sampling framework used for this document analysis: a stratified randomized sample of 70 municipalities representing the distribution across three municipality classification groups originally developed by the Swedish Association of Local Authorities and Regions (The Swedish Association of Local Authorities and Regions 2023): Group A (metropolitan municipalities and suburban municipalities), Group B (larger towns and surrounding areas), and Group C (smaller towns and rural municipalities). In addition to the 70 randomized municipalities, Sweden’s three metropolitan municipalities were included.

**Figure 1:**
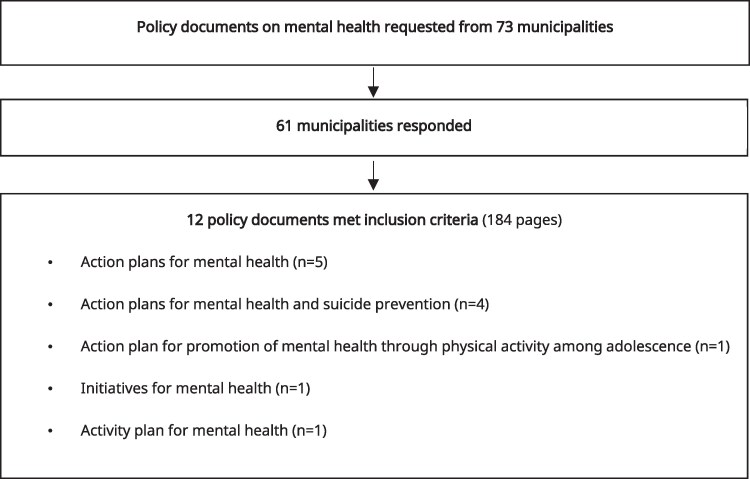
Selection of policy documents.

A municipality-wide mental health policy document was requested by e-mail from the 73 municipalities. The e-mail specified that the requested document should be a municipality-wide policy in use in 2024, focusing on mental health or on both mental health and suicide prevention. Municipalities that did not respond initially were sent one reminder. Sixty-one municipalities responded with some form of local policy document. To be included in the study, a policy document had to be municipality-wide and address mental health, or both mental health and suicide prevention. Policy documents that focused solely on suicide prevention were excluded, as were public health policies or social sustainability policies. Local collaboration agreements between the municipality and the regional health authority regarding individuals with mental health disorders were excluded. Following this selection process, 12 policy documents from 12 different municipalities remained for analysis (see Fig. 1). These municipalities represented all three municipality classification groups and Sweden’s three major regions (Götaland, Svealand, and Norrland). The final sample included one metropolitan municipality and two suburban municipalities (Group A), two larger municipalities (Group B), and seven smaller municipalities (Group C).

### Data analysis

**Figure 2:**
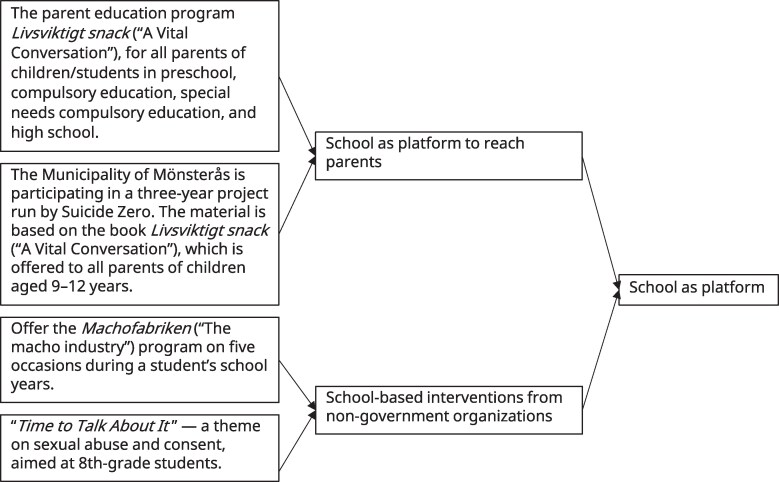
Example of grouping of codes and subcategories using conventional content analysis (Hsieh and Shannon 2005).

A document analysis was conducted following the four steps of the READ approach (Dalglish *et al*. 2020): (i) ready materials, (ii) extract data, (iii) analyze data, and (iv) distill findings. After collecting the policy documents, they were read several times to gain an overall understanding and all data relevant to children and adolescents were extracted during this reading. As recommended by the READ approach (Dalglish *et al*. 2020), a specific analysis methodology was applied; a conventional content analysis (Hsieh and Shannon 2005) was used. The extracted data were coded using the program NVivo (version 15) to identify words or phrases that captured central ideas relevant to the school’s role in mental health promotion and prevention. Similar codes were grouped into subcategories, which were further organized into broader categories to structure the findings (see Fig. 2 for an example). This was followed by further reading and deeper engagement with the literature in the field. Thereafter followed an iterative process in which the categories were refined in relation to central concepts in the field, with the aim of deepening the understanding of the collected data. All authors, who have backgrounds in psychology, public health, and social work, reviewed and agreed on the final categories. To ensure transparency, illustrative empirical examples that typically captured and represented the content of each category were selected from the policy documents (Bryman 2016).

### Ethical approval

The study was approved by the Swedish Ethical Review Authority (reg. no. 2025-04457-01), which determined that it is not covered by the Swedish Ethical Review Act, as the study does not involve the processing of sensitive personal data or any interaction with living research participants. The Swedish Ethical Review Authority raised no objections to conducting the study.

## Results

Schools were mainly approached in relation to compulsory education (ages 6–16), although high school (ages 16–19) was also included in the majority of the policy documents. Four categories were constructed: *the school as a context influencing mental health*; *the school as a platform for reaching all children and adolescents with mental health interventions*; *the school as responsible for detecting mental health problems*; and *the school as a collaborating partner*. The content within the categories covered similar areas, yet the activities they encompassed differed substantially. This was particularly evident in the first two categories, whereas the activities in the latter two categories were less diverse.

### The school as a context influencing mental health

Schools were consistently highlighted as a key context for the promotion of well-being and the prevention of mental health problems among all children and adolescents. The school environment was framed as important for shaping mental health outcomes, both through education and as a setting in which children’s health behaviors can be influenced. Consequently, the mental health of all children and adolescents was considered to be affected by the design of the school environment. A discrepancy was noted between how the school was described in general terms and the activities planned for implementation. Almost all policy documents emphasized the relationship between academic achievement at the end of compulsory education and future mental health outcomes when describing the school’s overarching role in promoting well-being and preventing mental health problems. As illustrated by the following empirical example, academic achievement was framed as protective factor for mental health in the policy documents.

All students should leave compulsory school and upper secondary school with passing grades. Achieving a passing result in school is a strong factor for good health later in life. (Tanum)

Consequently, attending school was described as important for mental health, as seen in the illustrative example below.

The foundation for leaving school with passing grades is to attend and participate in the lessons. (Gotland)

When the school was conceptualized as a context that itself influences mental health, schools were expected to develop environments that promote well-being and prevent mental health problems. How this was to be achieved varied considerably across the policy documents, with different activities emphasized in efforts to create a mental health-promoting context. One such approach involved increasing physical activity during the school day to promote well-being and prevent mental health problems.

Promote physical activity by working on the design of the school environment, including playgrounds, common areas, after-school activities, and more. (Stockholm)

Another way was to target the learning environment, for example, through the evidence-based PAX program, which aims to enhance safety and create a conducive learning environment in the classroom (The National Board of Health and Welfare 2025). To provide a safe learning environment was thus considered as a strategy to promote mental health, as seen in the following example.

Implement the evidence-based PAX method in schools to promote mental health. (Huddinge)

Safeguarding a health-promoting learning environment could also be achieved by leveraging the expertise of school health services in school development. To include school health service as a resource for over-all school development was thus framed as a way to promote both learning and health in the policy documents.

School health services are a resource for health-promoting school development and, together with school staff, are intended to contribute to creating environments that support students’ school attendance, learning, development, and health. (Helsingborg)

Responsibility for matters concerning the school as a context influencing mental health was assigned to the municipality as a whole, the school administrations, and, to some extent, ‘the school,’ and the school health services.

### The school as a platform for reaching all children and adolescents with mental health interventions

The school was approached as a platform in the majority of the policy documents. In this role, it served as an arena where all children, adolescents, and their parents could be reached directly with school-based initiatives targeting mental health. There was considerable diversity in the activities described across the policy documents. The structured school-based interventions outlined in the policy documents were primarily psychoeducational in nature. Their main focus was on enhancing children’s and parents’ knowledge about mental health, increasing awareness of health-related behaviors linked to mental well-being, and strengthening individual capacities to cope with life challenges. Some activities involved structured interventions, such as evidence-based programs or nationally guided programs administered by the school nurse meeting all children individually for health dialogues. Youth Aware of Mental Health (YAM) was a commonly mentioned evidence-based program in the policy documents.

All students in grade 8 receive training in YAM (Youth Aware of Mental Health), which aims to develop students’ skills to cope with life’s challenges and increase knowledge about mental health. (Tanum)

Many of the municipalities also offered locally developed school-based solutions or used material developed by non-governmental organizations. The example below illustrates one of the many different ways in which locally developed solutions to improve mental health and well-being was described in one of the policy documents.

In Söderköping Municipality, school counselors and school nurses also work with the programs *Hälsa Mående* (Health State of being) and *Säkra varje elev (Secure every student)*. These are models that allow for a structured review of various aspects related to mental health and well-being, with different areas of focus depending on the grade level. At the high-school level, a model called *Älska/Orka (Love/Manage)* is used, which corresponds to *Hälsa Mående (Health State of being)*. (Söderköping)

In some municipalities, like in the example below, the schools were also used as a platform to reach parents of children and adolescents with parental education to strengthen their children’s mental health.

Continued implementation and development of universal parenting support for parents of teenagers, using the school as an arena. (Hjo)

Although some efforts in this category were at the indicated level, the activities approaching the school as a platform were primarily focused on promotive and universal preventive levels. School health services were largely responsible for the interventions. The interventions in this category were only loosely connected to the educational context or mission of the school. Instead, mental health was addressed as an independent issue, rather than being linked to factors that might contribute to mental health problems, such as family issues, peer relationships, or academic pressure.

### The school as responsible for detection of mental health problems

Another way in which schools were approached in the policy documents was as an actor responsible for detecting mental health problems. This was described both in general terms and with respect to certain risk groups, but always with a focus on identifying the individual. When early detection was described more generally, the school was often portrayed as one of many actors in the community sharing this responsibility. The following example illustrates how the school’s role in detecting mental health problems could be formulated.

The school also has an important role in identifying children who may need support in their role as next of kin. (Gotland)

Activities related to the detection of mental health problems were often linked to evidence-based suicide prevention programs designed for school personnel. In contrast to evidence-based interventions directly aimed at children to strengthen their mental health-related skills, such as YAM in the previous category, these activities focused on enhancing staff members’ ability to identify and respond to mental health problems among children and adolescents. *‘Stör Döden’* (Disturb death) was mentioned in several policy documents as a method to help school staff detect mental health problems among students, as seen in the example below.

*‘Stör Döden’* (Disturb death) is a training program designed to provide teachers and school staff with support in working with students’ mental health. The training is entirely digital, and an individual teacher or staff member can complete it independently, thereby hopefully gaining new ideas, knowledge, strategies, and greater confidence in addressing students’ mental health. (Helsingborg)

Responsibility for schools’ overarching role in detecting mental health problems was primarily assigned to the municipalities and school administrations. Teachers, school health services, and school personnel more broadly were all portrayed as central to the practical implementation of mental health problem detection.

### The school as a collaborating partner

Lastly, the school was approached as a stakeholder responsible for collaborating on interventions for individual children with mental health problems, including defining roles and responsibilities around the child. The school shared this responsibility with other stakeholders within the municipality, such as social services, as well as with stakeholders outside the municipality, such as child and adolescent psychiatry. Collaboration largely focused on the indicated level, that is, coordination around students with identified mental health problems. Only a few policy documents described collaboration in promotive and preventive initiatives.

When collaboration was discussed in broader terms, the focus was ensuring that the child received the right intervention in time. Collaboration around a child with mental health problems was consistently framed as a non-negotiable requirement for all municipal actors, including schools, as exemplified below.

Children and adolescents with mental health problems should receive the right interventions at the right time through consolidated expertise. Children and adolescents who require coordinated interventions should have an SIP (*Samordnad individuell plan* [coordinated individual care plan]). (Lilla-Edet)

In contrast to the clear expectations regarding collaboration, the activities in this category were described in more abstract and vague terms. The emphasis was on fostering mutual understanding of respective roles and on developing more general forms of collaboration. The following illustrative example shows how this was formulated in one of the policy documents.

Through BUS (*Barn och unga i samverkan* [Collaboration for Children and Youth]), develop and implement working methods to increase knowledge of each other’s and the region’s roles and responsibilities, in order to connect individuals to appropriate support. (Huddinge)

Collaboration was expected to take place at all levels, from top management to professionals working directly with children and adolescents, although no specific profession was identified as responsible. Instead, school administrations, together with other administrations, were ultimately responsible for collaboration.

## Discussion

The aim of this study was to explore how the role of schools is approached in Swedish municipal policy documents governing mental health efforts. The findings showed that schools were approached in several ways: as a context influencing mental health, as a platform for reaching all children and adolescents with mental health initiatives, as an actor responsible for detecting mental health problems, and as a collaborating partner responsible for working with other stakeholders around individual children with mental health problems. While the first two categories were mainly universal and included both promotive and preventive efforts, the latter two were primarily at the indicated level (Institute of Medicine Committee on Prevention of Mental Disorders 1994). Given the extent to which schools were addressed in the policy documents, this study confirms previous research findings (Fjellfeldt and Eriksson 2026) and reflects national policy (The Public Health Agency of Sweden 2022, Government Offices of Sweden 2025), in which schools are positioned as key actors in Swedish municipalities’ promotive and preventive mental health efforts.

At first glance, the results of this study seem to suggest that schools are viewed as both settings for mental health promotion and platforms for implementing local mental health initiatives (World Health Organization 2021). When the school was approached as a context influencing mental health, the policy documents emphasized eligibility for and completion of high school as crucial protective factors for future mental health development. Additionally, developing a learning environment that influences mental health was highlighted as important. These strategies are supported by both international and national public health policy (World Health Organization 2018, The Public Health Agency of Sweden 2022), as well as by empirical research (Gustafsson *et al*. 2010, Partanen 2019, Högberg 2021, 2024, Jourdan *et al*. 2021, Klapp *et al*. 2024). However, a closer examination of the policy documents reveals that teachers, one of the cornerstones of the health-promoting school (St Leger 2005, Jourdan *et al*. 2021), were largely absent from policy documents when the school was approached as a context influencing mental health. Rather than focusing on how teacher–student relationships (Modin *et al*. 2011, Brolin Låftman *et al*. 2023) and pedagogical strategies (Bortes and Giota 2024, Bortes 2025) can shape both mental health outcomes and academic performance, the policy documents treated the learning environment as an influencing factor independent of teachers and educational support. Specific measures emphasized in Swedish national public health policy (The Public Health Agency of Sweden 2022) and the Education Act (Skollag, 2010:800) such as ensuring a sufficient proportion of teachers with teaching degrees, early identification of learning difficulties (Education Act 2010, The Public Health Agency of Sweden 2022), and special educational support to improve academic achievement (Education Act 2010, The Public Health Agency of Sweden 2022) were all absent from the policy documents.

This omission is particularly concerning because school-related stress, stemming from high academic demands combined with insufficient educational support, is one of the main contributors to mental health problems in Sweden (Löfstedt *et al*. 2020, Högberg *et al*. 2021, Högberg 2024, Klapp *et al*. 2024). Consequently, the local policy documents lacked strategic guidance on how to achieve the school’s educational mission. Teachers, teaching quality, and access to pedagogical support were not conceptualized as integral components of promoting well-being and preventing mental health problems. In short, when schools were viewed as a context influencing mental health, the policy documents failed to target one of the main social determinants of mental health within the school system—education.

How the school functions as a social arena is an equally important determinant of mental health development for children and adolescents within schools (Modin *et al*. 2017, Modin 2021). However, this critical aspect of the school’s role in mental health promotion and prevention was also notably absent when schools were approached as contexts influencing mental health. Social relationships between teachers and students (Yu *et al*. 2022), peer relationships (Brolin Låftman *et al*. 2023), school connectedness (Diggs *et al*. 2025), and a school that counteracts bullying (Modin *et al*. 2015, Ringdal *et al*. 2020) are all crucial factors in regard to the school’s role in mental health promotion and prevention. The substantial contribution of schools as social contexts thus represents another gap in the local mental health policy documents examined, seemingly lost in the local implementation of international policy (World Health Organization 2018, Jourdan *et al*. 2021), Swedish public health policy (The Public Health Agency of Sweden 2022), and the Swedish curriculum (The Swedish National Agency for Education 2024a), all of which emphasize the importance of the social environment.

When the school was approached in its role as a platform, the activities were described in considerable detail. In this role, the school was primarily seen as a means of reaching all children and adolescents directly through universal, mainly psychoeducational, interventions targeting students and their parents. School health services were largely responsible for these interventions. This reflects a traditional approach to school health promotion, using the school as an arena for health professionals to provide information (Rowling and Jeffreys 2006, Jourdan *et al*. 2021, Hernández-Torrano *et al*. 2024). While this aligns with international policy advocating the use of schools to strengthen individual health literacy (World Health Organization 2018, 2021), it also reflects biomedical conceptualization of mental health (Jourdan *et al*. 2021). These results are consistent with some of the findings of Elsayed *et al*. (2023), who showed that the health promotion discourse in local Swedish educational policies was partly predominated by a biomedical discourse, marginalizing teachers in favor of healthcare professionals by virtue of being ‘scientific and expert’ (Elsayed *et al*. 2023).

Furthermore, a growing body of research on universal school-based mental health promotion and prevention raises concerns. While evidence is more conclusive in support of selective and indicated interventions in schools (Pilling *et al*. 2020, Guzman-Holst *et al*. 2025, Spencer *et al*. 2025), large, well-designed studies have reported mixed results, including no effects and, in some cases, negative effects for universal psychoeducational programs (Foulkes *et al*. 2025, Guzman-Holst *et al*. 2025). In addition, it has long been recognized that many of these programs struggle with implementation, uptake, and sustainability. A well-known challenge in implementing mental health prevention programs is that they do not easily adapt to the local context (Rowling and Jeffreys 2006, Jourdan *et al*. 2021). This raises the question of how the encouragement to implement universal mental health interventions should be understood given their known limitations.

An overarching finding was the considerable variation in how municipalities expected schools to engage in mental health–promoting and preventive activities, particularly when viewing the school as a context and as a platform. This highlights that, despite the recognized importance of schools in national policy (Education Act 2010, The Public Health Agency of Sweden 2022, Government Offices of Sweden 2025), their role in mental health efforts is subject to local interpretation. These findings align with Danish studies showing that, while overall educational aims are set centrally, responsibility for planning health education lies with local authorities and schools, leading to fragmented and inconsistent health promotion initiatives (Simovska 2012, Lindegaard Nordin *et al*. 2019). In this study, the scattered activities found in local mental health policy documents may reflect the decentralization of the school system (The Swedish Association of Local Authorities and Regions 2025a). Furthermore, the finding is consistent with those from other studies on the local implementation of Sweden’s national governance of student health services. These studies have suggested that differing interpretations at local level may stem from lack of specificity in national policy (Bergnehr and Johansson 2023, Hammerin *et al*. 2023, Elsayed 2025). Hence, the possibility for various local interpretations may weaken the potential and significance attributed to schools in international and national public health policy. The level of school governance and policy design may therefore be factors that hinder or enable a coherent national approach regarding the role of schools in promoting and preventing mental health problems among children and adolescents.

In comparison to the varying ways in which schools were approached as a context and platform in universal mental health efforts, the policy documents were more consistent in outlining the school’s responsibilities at the indicated level. Schools were tasked with detecting and addressing mental health problems, as well as collaborating with other stakeholders to coordinate interventions for individual children with mental health problems. These goals and activities may reflect local agreements with regional psychiatric healthcare, financially incentivized by the Swedish government to stimulate cross-sectoral collaboration between regions, which govern healthcare, and municipalities in local mental health promotion and suicide prevention efforts (The Swedish Association of Local Authorities and Regions 2025b). Detecting, addressing, and coordinating interventions for children and adolescents with mental health problems is an important responsibility for schools, as it is crucial from both an ethical and life-course perspective (Costello 2016, Svensson and Warne 2024).

At the same time, the clear and consistent emphasis on detection and individual support in collaboration with psychiatry situates both the concept of mental health and the school’s role in mental health efforts within a healthcare context. Scholars argue that a medicalized understanding of mental health problems may overlook the everyday challenges to which children and adolescents emotionally respond, and in doing so, risk translating social and structural issues into individual psychological terms (Due *et al*. 2019, Wickström and Lindholm 2020, Wickström and Zeiler 2021, Eriksson and Stattin 2024, Stattin *et al*. 2025). While a biopsychosocial perspective in school health promotion acknowledges that mental health is also shaped through interactions with health determinants within the school environment, a medical understanding of mental health emphasizes the involvement of healthcare professionals (Jourdan *et al*. 2021). By focusing the role of schools on detecting, addressing, and collaborating around individuals with mental health problems, the local mental health policy documents frame these problems as originating within the student. This, in turn, partially shifts the responsibility for mental health problems from the school setting to the healthcare system.

### Strengths and limitations

While there are many studies exploring the implementation of mental health policies in schools (e.g. Guerra *et al*. 2019, Teutsch and Gugglberger 2020), this study provides novel insights through its unique design. By directing the analytical focus toward local mental health policy documents, the study enables an examination of the role of schools from an external perspective, highlighting how schools are understood and positioned within broader local mental health efforts. However, there are limitations to this study. First, despite a high response rate (89%) from the randomized municipalities, only a limited number of policy documents (12) met the inclusion criteria. More overarching municipal public health policies and social sustainability plans were excluded as the aim of this study was to explore how the role of school is approached in municipal policy documents specifically governing mental health efforts. Second, municipal policy documents on mental health represent only one type of local document in which schools might be assigned responsibilities. The aim of the policy inevitably shapes the ways in which the school’s role is framed and understood. For instance, the results of this study partly contrast with those from a discourse analysis of school health promotion in Swedish national and municipal educational policies, where an overt focus on learning and educational outcomes was detected (Elsayed *et al*. 2023). The choice of policy document may risk narrowing the understanding of the school’s role in mental health promotion and prevention. Nevertheless, the chosen policy documents efficiently capture the role of schools in governing local mental health efforts in Sweden. Third, the policy documents may not accurately reflect actual practices within municipalities, as gaps between policy and implementation may exist (Hupe and Hill 2016).

### Implications for future research

Building on the findings of the present study, future research should examine how these policy expectations are put into practice in Swedish schools. For example, studies could explore how school professionals experience their work in mental health promotion and prevention. In addition, research focusing on students’ perspectives would contribute important knowledge about how school mental health promotion and prevention is experienced. Further research could also investigate how similar policy constructions of schools’ roles are articulated and implemented in other national or local policy contexts.

## Conclusions

This study contributes to the research on school health promotion by providing an in-depth understanding of how the role of schools is approached in Swedish municipal policy documents governing mental health efforts. Overall, the analysis highlighted a predominantly individualized and often medicalized understanding of mental health problems when schools were viewed as platforms for reaching all children with interventions, as responsible for detecting mental health problems, and as responsible for collaborating with other stakeholders around children with mental health problems. These roles positioned school health professionals as key actors in delivering universal school-based mental health interventions and referring students to treatment outside of schools. At the same time, the role of schools as a context influencing mental health was clearly acknowledged. However, teachers and pedagogical support, cornerstones of health-promoting schools and a settings-based approach, were absent from this role. Further, the important contribution of schools as social arenas represented another notable gap in the policy documents examined. This suggests that the school’s role in mental health efforts was largely detached from its educational mission in the policy documents. Hence, although school success was recognized as important the local policy documents partly fail to address a key social determinant of mental health within the school system: educational conditions. Finally, there was substantial variation in the activities expected of schools across municipalities. Hence, despite the recognized importance of schools in public health policy, their role in mental health efforts is subject to local interpretation of national policy within a decentralized school system. The level of school governance and the design of policy may therefore be important to the ability to implement a coherent national approach to promoting and preventing mental health problems among children and adolescents.

## Data Availability

All policy documents reviewed in this study are public records; those interested in the underlying material may contact the corresponding author.
